# Intact PCL is a potential predictor of ACL graft size in the skeletally immature knee and other anatomic considerations for ACL reconstruction

**DOI:** 10.1186/s40634-021-00437-9

**Published:** 2022-01-06

**Authors:** David M. Heath, Alexander V. Nguyen, Travis S. Bullock, Samuel S. Ornell, Katherine C. Bartush, Grant D. Hogue

**Affiliations:** 1 Department of Orthopaedics, UT Health San Antonio, San Antonio, TX 78249 USA; 2 Department of Orthopaedics, Lenox Hill Hospital, New York, NY 10075 USA; 3 Department of Orthopaedics, Boston Children’s Hospital/Harvard Medical School, 300 Longwood Avenue, Boston, MA 02115 USA

**Keywords:** Pediatric, Knee, Anterior cruciate ligament, ACL, Posterior cruciate ligament, PCL, Arthroscopy, Magnetic resonance imaging, MRI, ACL reconstruction

## Abstract

**Purpose:**

To develop a method for using an intact posterior cruciate ligament (PCL) as a predictor of anterior cruciate ligament (ACL) graft size and examine possible differences in tunnel length based on all-epiphyseal drilling method.

**Methods:**

One hundred one patients 5–18 years of age with magnetic resonance imaging (MRI) of the knee at an outpatient pediatric orthopaedic clinic from 2008 to 2020 were included. ACL and PCL coronal, sagittal, and length measurements were made in all patients. Tunnel length measurements were made in patients with open physes. Statistical analyses were performed to evaluate potential associations in patient bony or ligamentous measurements.

**Results:**

PCL sagittal width and PCL coronal width were statistically significant predictors of ACL sagittal width and ACL coronal width, respectively (*p* = 0.002, R = 0.304; *p* = 0.008, R = 0.264). The following equations were developed to calculate ACL coronal and sagittal width measurements from the corresponding measurement on an intact PCL; ACL Coronal Width (mm) = 6.23 + (0.16 x PCL Coronal Width); ACL Sagittal Width (mm) = 5.85 + (0.53 x PCL Sagittal Width). Mean tibial maximum oblique length (27.8 mm) was longer than mean tibial physeal sparing length (24.9 mm). Mean femoral maximum oblique length (36.9 mm) was comparable to mean femoral physeal sparing length (36.1 mm). Both were longer than mean femoral straight lateral length (32.7 mm).

**Conclusion:**

An intact PCL is a predictor of native ACL size. Tunnel length differs based on chosen drilling method in all-epiphyseal technique.

**Level of evidence:**

Diagnostic Level III.

## Introduction

Anterior cruciate ligament (ACL) injuries in pediatric patients were historically treated with conservative measures until skeletal maturity to avoid iatrogenic physeal injury and subsequent effects on growth [1, 6]. However, such delays in surgery have been shown to be associated with poorer functional outcomes and a higher risk of cartilage or meniscal injury [1, 4, 6, 8, 13]. As the rate of ACL reconstruction in skeletally immature patients has increased, surgeons have developed techniques aimed at restoring knee stability while simultaneously minimizing risk to meniscal and physeal structures [1, 6].

With animal studies demonstrating the percentage of volumetric injury to the physis to increase with larger diameter drill holes and with more oblique tunnels, physeal sparing or all-epiphyseal operative techniques have been developed to minimize risk to the physis and growth arrest [9, 12]. Still, the distal femoral and proximal tibial growth plates have complex anatomic morphologies that continue to change with patient age, highlighting the importance of understanding the relationship between patient morphology and patient demographic to minimize the risk of physeal injury intraoperatively [3, 5, 14].

Prior studies have examined the relationship of patient age, sex, and height with ACL morphology, finding ACLs to generally increase in length and cross-sectional area, and to become more vertical in the sagittal and coronal planes as patients age [7, 8]. While ACL growth occurs during a predictable age range, the specific ACL length and cross-sectional area are significantly different between males and females with increasing patient height [13, 15]. Given the reports of differences in ligamentous morphology based on demographic characteristics, it is imperative to further explore all patient factors contributing to ligamentous variability. Understanding the anatomy of the skeletally immature knee remains at the forefront of surgical planning for patient safety and surgical efficacy during ligamentous reconstruction. Further knowledge of patient-specific factors that may influence this morphology in a predictable manner could benefit orthopaedic surgeons in avoiding iatrogenic physeal injuries while also improving patient outcomes.

This study aims to further current pediatric orthopaedic knowledge by investigating reproducible measurements of knee anatomy. The primary outcome was to examine if intact PCL measurements serve as a potential predictor of ACL graft size by approximating the native ACL. The secondary outcomes sought to introduce the concept of ACL:PCL dimensional ratios and examine potential associations with patient age, height, sex, weight, race, and ethnicity. Potential differences in tunnel length with differing tunnel locations on both the femoral and tibial sides based on different tunnel locations with respect to the physis were also investigated.

## Materials and methods

**Fig. 1:**
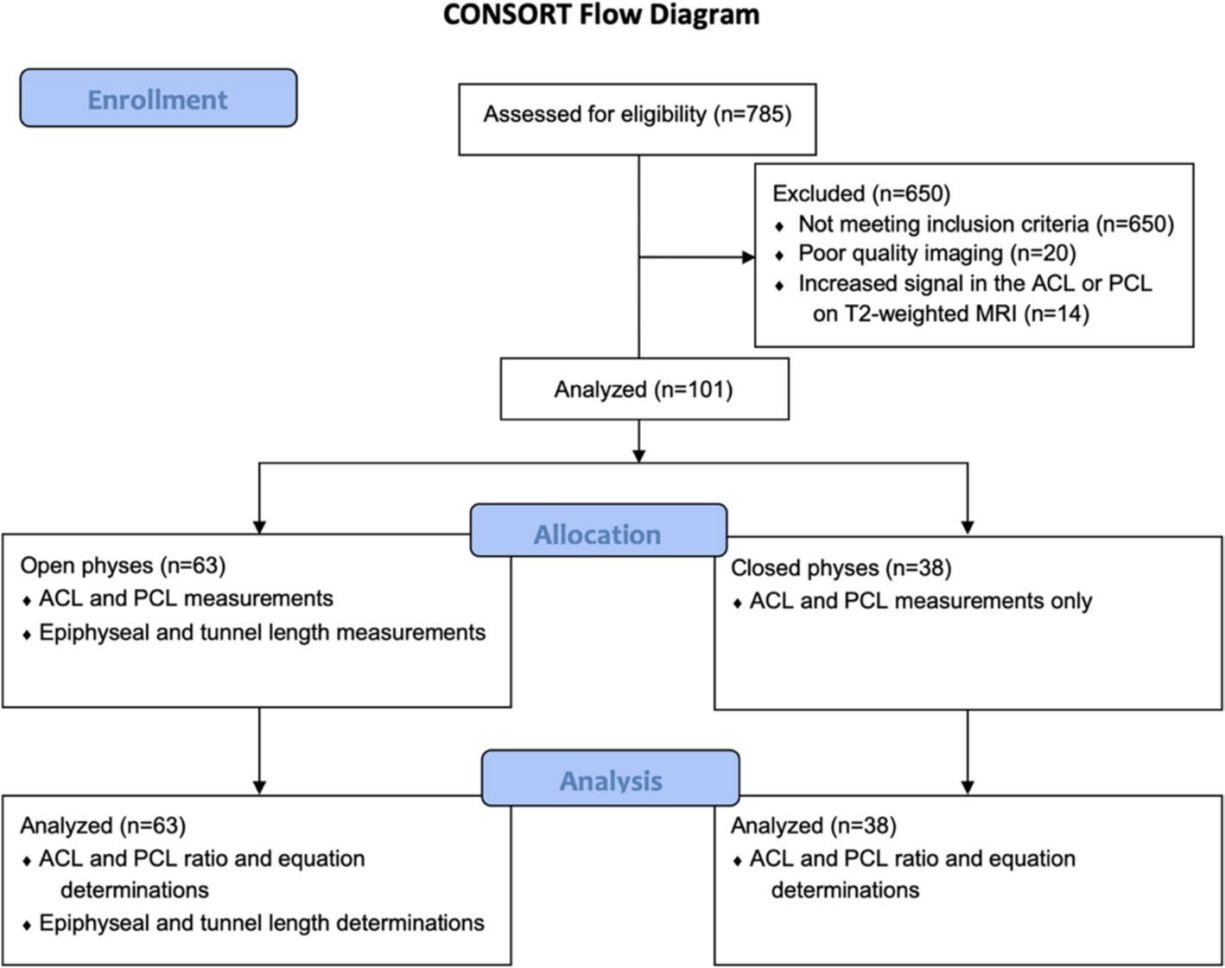
Subject selection process. CONSORT flow diagram detailing each step of inclusion and exclusion criteria with subject numbers included

The protocol for this study was approved by the Institutional Review Board (IRB) of institution at which it was performed. There was no external source of funding. The research team was comprised of three orthopaedic residents, one orthopaedic sports medicine fellow, one orthopaedic sports medicine faculty, and one pediatric orthopaedic faculty member. Retrospective electronic medical record (EMR) review identified patients 5–18 years of age with clinical orders for magnetic resonance imaging (MRI) of the knee at an outpatient pediatric orthopaedic clinic in San Antonio, Texas from 2008 to 2020. Inclusion criteria were patients 5–18 years of age with a completed, reviewable MRI of the knee and no prior knee surgery. Exclusion criteria were increased signal in the ACL or PCL on T2-weighted MRI, other evidence of ACL or PCL tear, prior knee surgery, incomplete data available for collection, or poor-quality imaging. The subject selection process is illustrated in the Consolidated Standards of Reporting Trials (CONSORT) flow diagram in Fig. 1. Three individual reviewers performed retrospective chart reviews and the measurements were strictly made as outlined in the following sections. Demographic information was collected on all patients from the EMR, including age in years at the time of their MRI, race, ethnicity, gender, height, weight, and laterality of the involved knee.

### Measurements

All measurements were made on T2-weighted MRI using the coronal and sagittal cuts in a.

standardized Picture Archiving and Communication System (PACS) image viewer. All measurements were rounded to the nearest millimeter (mm). The ACL and PCL sagittal and coronal widths were measured at the mid-substance of each ligament.

The following measurements were made on all patients:

1. ACL length, coronal width, and sagittal width
2. PCL length, coronal width, and sagittal width

In patients with open physes, the following measurements were made:

1. Height of tibial epiphysis from the anterior cortical rim to the physis
2. Tibial epiphyseal maximum oblique length, tibial physeal sparing length
3. Height of femoral epiphysis measured from the middle of the lateral femoral condyle at the joint to the femoral physis
4. Femoral epiphyseal maximum oblique length, femoral physeal sparing length, and femoral straight lateral length

**Fig. 2:**
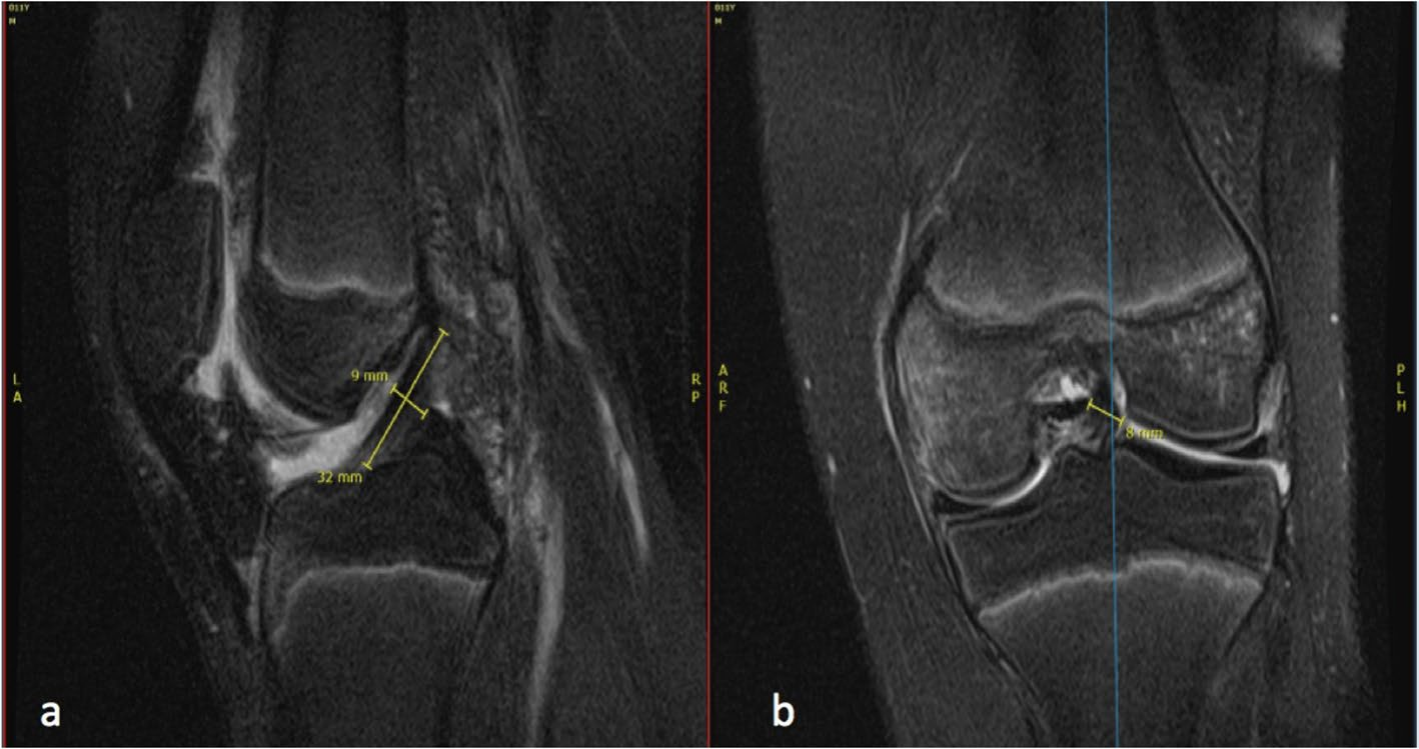
Example ACL measurements. **A** Sagittal ACL width (9 mm) and ACL length (32 mm). **B** Coronal ACL width (8 mm)

**Fig. 3:**
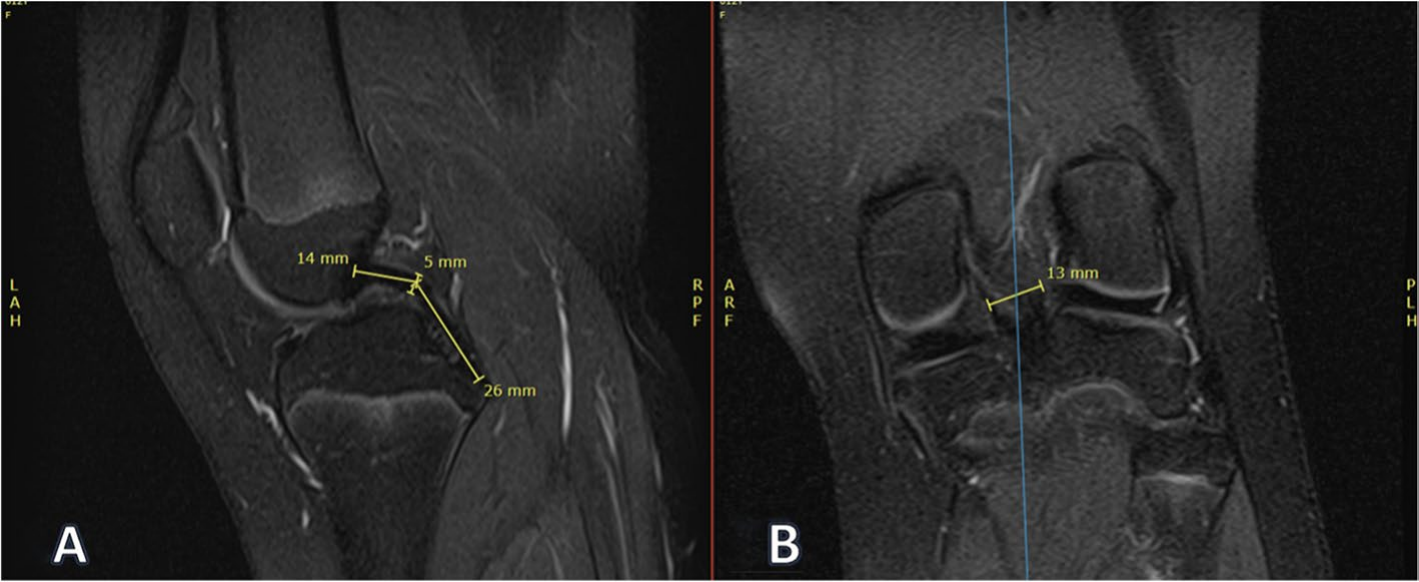
Example PCL measurements. **A** Sagittal PCL width (5 mm) and PCL length (14 mm + 26 mm = 40 mm). **B** Coronal PCL width (13 mm)

**Fig. 4:**
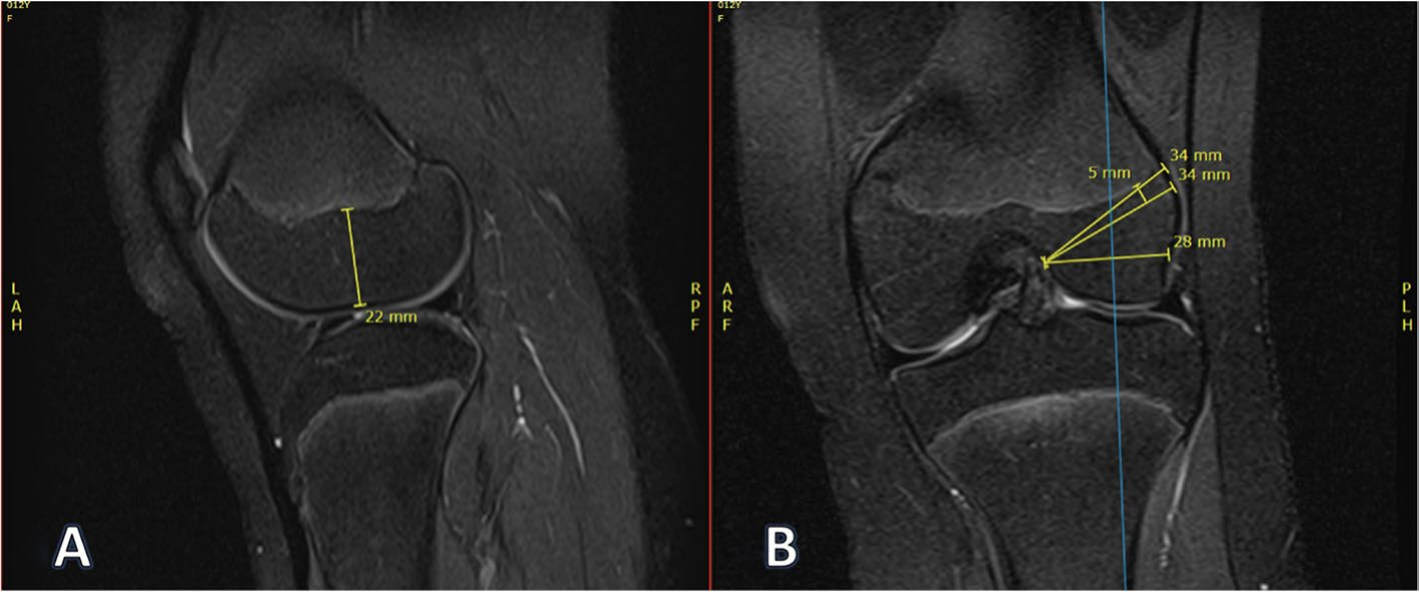
Example femoral epiphysis measurements. **A** Femoral epiphysis height (22 mm). **B** Femoral tunnels from top to bottom: maximum oblique (34 mm), physeal sparing (5 mm from physis, 34 mm), and direct horizontal (28 mm)

**Fig. 5:**
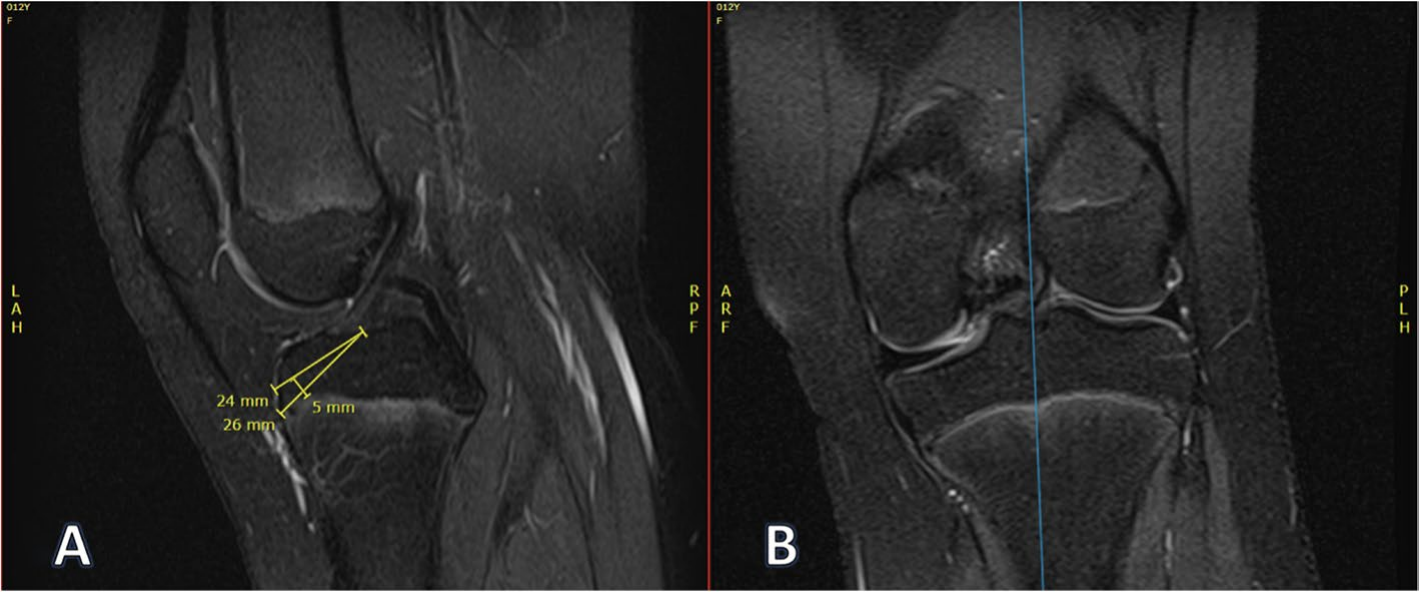
Example tibial epiphysis tunnel length measurements. **A** Tibial physeal sparing length (24 mm) and tibial maximum oblique length (26 mm). **B** Corresponding coronal location of measurements. These measurements were made on the most medial cut that displayed the ACL footprint

**Fig. 6:**
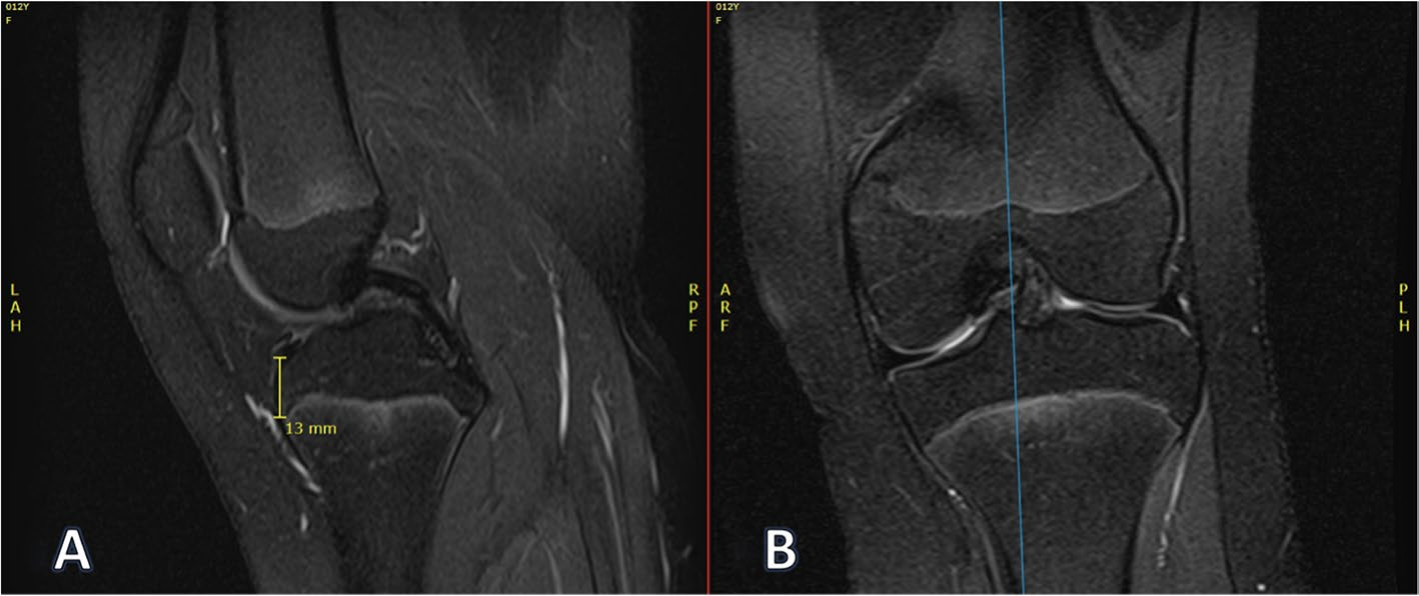
Example tibial epiphysis height measurement. **A** Height of tibial physis (13 mm). **B** Corresponding coronal location of measurement. This measurement was made just lateral to the medial tibial spine to approximate ACL tibial tunnel position

These measurements were made to represent potential tunnel locations for an all-epiphyseal.

or physeal-sparing ACL reconstruction technique and to examine if any difference exists.

between tunnel lengths. Example measurements are displayed in Figs. 2, 3, 4, 5 and 6.

The maximum tibial oblique length was measured in the sagittal view from the ACL footprint to the anterior tibial cortex without crossing the physis. The tibial physeal sparing length was measured from the ACL footprint to the anterior tibial cortex 5 mm from the level of the physis.

The femoral maximum oblique, physeal sparing, and straight lateral lengths were measured on the coronal view. The femoral maximum oblique length was measured from the ACL origin to the level of the physis on the lateral cortex of the femur. The femoral physeal sparing length was measured from the ACL origin to 5 mm below the physis level on the femur’s lateral cortex. The femoral straight lateral length was measured from the ACL origin to the lateral femoral cortex.

### Statistical analysis

All statistical analyses were performed using the Statistical Package for the Social Sciences (SPSS) software version 27.0 (IBM Corporation, Armonk, NY). Data are summarized as mean, standard deviation (SD), median, and range. An independent t-test was performed to evaluate the means of two categorical groups. A paired t-test was performed to evaluate patients undergoing multiple bone measurement comparisons, applying the Bonferroni correction where appropriate. Pearson’s correlation (R) and simple linear regression were performed to evaluate associations between two continuous variables. All tests were conducted at the alpha level of 0.05.

## Results

Initially, 785 patients were identified for inclusion in this study. After application of exclusion criteria, 101 patients were available for final statistical analyses. None of these patients had bilateral imaging. Sixty-three patients were determined to have open physes. Patient demographics are detailed in Table 1.

**Table 1:** Demographics

	N (%)	Mean (SD) Median (min, max)
Male	59 (58.4)	–
Female	42 (41.6)	–
Hispanic	69 (68.3)	–
Non-Hispanic	32 (31.7)	–
Caucasian	89 (88.1)	–
Non-Caucasian	12 (11.9)	–
Age (years)	101 (100)	15 (2) 15 (8, 18)
Height (cm)	86 (85.1)	167 (11.5) 168 (142, 190)
Weight (kg)	86 (85.1	75.4 (23.4) 71 (33, 137)

Positive correlations were found across all three ACL and PCL measurements. Linear regression analyses demonstrated that PCL sagittal width and PCL coronal width were statistically significant predictors of ACL sagittal width and ACL coronal width, respectively (*p* = 0.002, R = 0.304; *p* = 0.008, R = 0.264). PCL length showed a trend toward predicting ACL length but did not reach statistical significance (*p* = 0.076). Table 2 displays a comparison of ACL and PCL measurements.

**Table 2:** Cruciate Ligament Comparisons

Ligamentous Measurement	Mean (SD)	Median (min, max)	***P***-value*	R
ACL Length (mm)	30.2 (3.5)	31.0 (22, 37)	.076	.178
PCL Length (mm)	39.8 (3.7)	40.0 (31, 48)		
ACL Sagittal Width (mm)	8.9 (2.0)	9.0 (5, 14)	**.002**	.304
PCL Sagittal Width (mm)	5.8 (1.1)	6.0 (4, 9)		
ACL Coronal Width (mm)	8.2 (0.9)	8.0 (6, 12)	**.008**	.264
PCL Coronal Width (mm)	13.3 (1.7)	13.0 (10, 17)		

*Significance of PCL measurement as a predictor of ACL measurement

Linear regression analysis using ACL coronal or sagittal width as the dependent variable and PCL coronal or sagittal width as the independent variable allowed for development of two equations which may be used to calculate native ACL dimensions via an intact PCL. Since PCL length did not statistically predict ACL length, no equation was developed in regard to length.

The equation that allows for the use of an intact PCL coronal width measurement to predict the coronal width of the native ACL is:

$$\mathrm{ACL}\ \mathrm{Coronal}\ \mathrm{Width}\ \left(\mathrm{mm}\right)=6.23+\left(0.16\ \mathrm{x}\ \mathrm{PCL}\ \mathrm{Coronal}\ \mathrm{Width}\right)$$

The equation that allows for the use of an intact PCL sagittal width measurement to predict the sagittal width of the native ACL is:

$$\mathrm{ACL}\ \mathrm{Sagittal}\ \mathrm{Width}\ \left(\mathrm{mm}\right)=5.85+\left(0.53\ \mathrm{x}\ \mathrm{PCL}\ \mathrm{Sagittal}\ \mathrm{Width}\right)$$

**Fig. 7:**
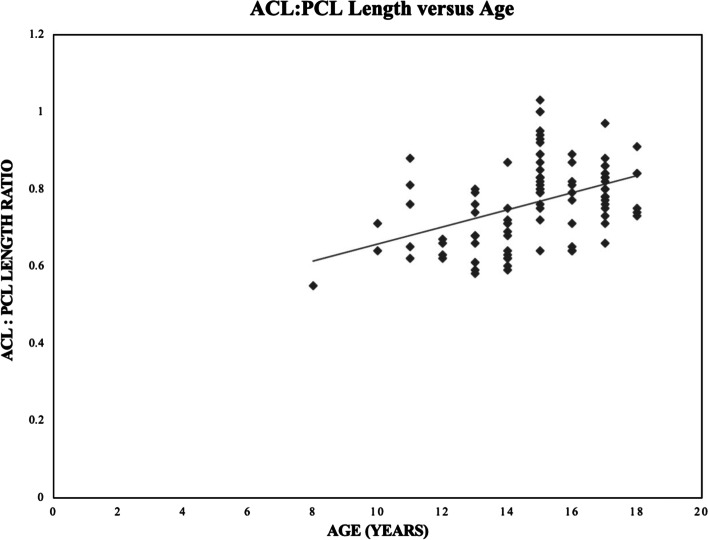
Scatter plot with best-fit line of ACL:PCL length ratio versus age in years. A positive correlation exists between ACL:PCL length ratio and age (*p* < 0.0001, *R* = 0.431)

The ratio of the size of the ACL to the size of the PCL in each plane, referred to in this study as the ACL:PCL ratio, was evaluated across patient demographics. Patient age was the only demographic that had an effect on any ACL:PCL ratio. Age was positively correlated with ACL:PCL length ratio (*p* < 0.0001, R = 0.431). A visual representation of this association is displayed in Fig. 7. ACL:PCL ratios by each demographic are presented in Table 3.

**Table 3:** ACL:PCL Ratios and Demographic Comparison

	ACL:PCL Length			ACL:PCL Sagittal Width			ACL:PCL Coronal Width		
	Mean (SD)	R	*P*-value	Mean (SD)	R	*P*-value	Mean (SD)	R	*P*-value
**Gender**									
Male (*n* = 59)	0.78 (0.09)		0.254	1.48 (0.38)		0.303	0.64 (0.09)		0.719
Female (*n* = 42)	0.75 (0.11)			1.58 (0.46)			0.63 (0.10)		
**Ethnicity**									
Hispanic (*n* = 69)	0.78 (0.10)		0.173	1.52 (0.43)		0.514	0.63 (0.10)		0.263
Non-Hispanic (*n* = 32)	0.76 (0.11)			1.39 (0.33)			0.66 (0.09)		
**Race**									
Caucasian (*n* = 89)	0.76 (0.09)		0.977	1.5 (0.39)		0.971	0.63 (0.09)		0.503
Non-Caucasian (*n* = 12)	0.76 (0.12)			1.51 (0.40)			0.65 (0.08)		
**Age**									
8–14 years (*n* = 37)	0.68 (0.07)	0.431	**< 0.001**	1.68 (0.43)	0.125	0.154	0.61 (0.08)	0.185	0.064
15–18 years (*n* = 64)	0.81 (0.09)			1.42 (0.37)			0.64 (0.10)		
**Height**									
142–167 cm (*n* = 40)	0.77 (0.11)	0.014	0.900	1.59 (0.42)	0.213	0.051	0.62 (0.09)	0.191	0.080
168–190 cm (*n* = 46)	0.76 (0.10)			1.47 (0.40)			0.63 (0.09)		
**Weight**									
33–70 kg (*n* = 45)	0.76 (0.10)	0.120	0.259	1.6 (0.38)	0.183	0.085	0.62 (0.09)	0.145	0.174
71–137 kg (*n* = 45)	0.77 (0.09)			1.44 (0.41)			0.64 (0.10)		

The mean tibial and femoral epiphyseal heights, mean tibial and femoral maximum oblique lengths, and mean tibial and femoral physeal sparing lengths are presented in Table 4. In comparing these bone measurements, all differences in the observed means reached statistical significance.

**Table 4:** Epiphyseal Measurements

Measurement	Mean (SD)	Median (min, max)	***P***-value
Tibial Epiphyseal Height (mm)	13.52 (2.32)	13.0 (10, 19)	**–**
Femoral Epiphyseal Height (mm)	26.82 (3.54)	27.0 (20, 39)	**–**
Maximum Tibial Oblique Length (mm)	27.8 (3.5)	28.0 (21, 35)	**<.001**
Tibial Physeal Sparing Length (mm)	24.9 (3.2)	25.0 (19, 32)	
Femoral Physeal Sparing Length (mm)	36.1 (3.6)	36.0 (29, 44)	**<.001**
Femoral Straight Lateral Length (mm)	32.7 (4.0)	32.0 (21, 42)	
Maximum Femoral Oblique Length (mm)	36.9 (3.6)	37.0 (29, 46)	

## Discussion

The most significant finding of this study is that PCL sagittal and coronal width measurements can be used to predict ACL sagittal and coronal width. Additionally, this is the first study to develop equations for predicting native ACL coronal and sagittal width from an intact PCL in the pediatric population. The results of this study indicate that there may be utility in using an intact PCL as a size predictor of the native ACL in patients presenting with ACL rupture, and also a way to apply this information in the clinical setting.

Moreover, ACL:PCL dimensional ratios and their relationship to demographic differences were examined, showing that only age contributed to the observable variance and solely in ACL:PCL length measurements in our patients. This finding is in line with previously published studies that have demonstrated ACL length to increase with age [7, 8]. The ACL therefore either outpaces the PCL in terms of length gained as pediatric patients age or it is not a linear relationship. A higher power study may elicit a more robust correlation, as the results of this study still indicate a potential predictive value in using PCL dimensional measurements for ACL estimates.

With the exception of the aforementioned effect of age on the ACL:PCL length ratio, the ACL:PCL ratio in all dimensional planes was not significantly different across gender, race, ethnicity, age, height, or weight. If there are no significant differences in ACL:PCL ratios across demographic groups and these ratios are potentially conserved, then utilizing these ratios may allow for a simplified and efficient calculation of graph size across an anatomically diverse patient population. The preservation of these ratios validates the use of the previously provided equations for calculating ACL coronal and sagittal width across different demographics. This further substantiates the potential for using an intact PCL as a predictor for the size of the native ACL in determining graft size for ACL reconstruction in the skeletally immature patient. However, this study only aims to raise this as a possibility as it is solely anatomic in nature. A follow-up study implementing this technique is necessary to determine its efficacy.

While advancements have been made in pediatric ACL reconstruction techniques, avoiding physeal injuries remains complicated by anatomic variations in the skeletally immature knee that may influence appropriate graft size selection. Prior studies have demonstrated growth disturbances occurring when 7% of physeal cross-sectional is disrupted [11]. For patients 10–15 years of age, drilling 8 mm tunnels in the tibia and femur is associated with 2.5% and 2.4% of physeal cross-sectional distruption respectively, with every 1 mm increase in tunnel diameter being associated with a 1.1% increase in volumetric injury [9]. Oblique tunnels were also shown to cause more significant volumetric injury than vertical tunnels and that volumetric injury decreased from 4.1% to 3.1% when the drilling angle was increased from 45 degrees to 70 degrees [9]. These figures indicate that it may be safe to drill an 8 mm tunnel in a skeletally immature knee without compromising the physis. But emphasize the importance understanding the relationship between patient morphology and patient demographic to minimize the risk of physeal injury intraoperatively. Some surgeons prefer avoidance of the physis altogether or prefer to drill smaller tunnels in young patients in an effort to further minimize risk of injury to the physis. In young patients who have undergone ACL reconstruction, their graft must endure as they continue to grow. The intra-articular portion of the graft has been demonstrated to increase in length, but not diameter during this period of continued growth [2, 10].

This study contributes to the existing literature by giving a greater understanding of how different tunnel locations affect tunnel length and potentially the overall length of the graft construct. Statistically significant differences were found between the tibial maximum oblique lengths and physeal sparing lengths. Although the femoral physeal sparing and maximum oblique lengths were similar, the overall length of the construct would change since the tibial tunnels were of different sizes. This would especially hold true if a straight lateral femoral tunnel was to be used since it was significantly shorter than the other two femoral tunnel methods. These statistical findings should be interpreted with clinical or operative relevance in mind, as the mean difference was 2.9 mm in the tibial measurements and 0.8 mm in the mean femoral measurements. If opting for the all epiphyseal ACL reconstruction technique, the physeal sparing tunnel location allows the surgeon to avoid the physis altogether without a change in tunnel length. These differences in tunnel lengths may need to be accounted for in preoperative planning in certain techniques to avoid a mismatch in the length of the tunnel and the graft.

The presented study is not without limitations. As multiple researchers participated in the measurement process, there is likely a component of interobserver variability. This effect was minimized by having only three involved observers, with each following a standardized protocol. Additionally, measurement specificity was limited by the PACS viewing software, which rounded measurements to the nearest millimeter. While this reduces the accuracy of the measurements, it is consistent with many systems in clinical use today and thus does not significantly affect the clinical applicability of the study. Since this is an MRI-based anatomical study utilizing T2 coronal and sagittal reconstructions, the clinical applicability of this study is both one of its greatest strengths and weaknesses. Preoperative planning can be carried out on the same imaging used to diagnose ACL tears without additional imaging, however this method is unvalidated since the study is purely anatomic. While the use of MRI studies with intact ACL and PCL ligaments was necessary for the study, it is not yet validated in the presence of an ACL tear. Although an uninjured PCL is unlikely to change in size after rupture, a confirmatory study in patients with MRI of intact cruciate ligaments prior to rupture of the ACL as well as MRI following ACL injury would allow measurement of the PCL in both scenarios to ensure there is no difference. The external validity of this study is limited by the patient demographics, which are influenced by geographic location. The demographics in this study were heavily skewed toward the white/Caucasian race (87.1%) and Hispanic ethnicity (68.3%). There were only ten patients of African descent (9.9%) and two patients of Asian descent (2.0%). Another limitation was the power of the study. While this is a large study in the context of a pediatric MRI-based anatomic study, future higher-powered studies are indicated to identify stronger correlations or dispute the presented findings. Lastly, the average patient age was 15 years. Many would consider using transepiphyseal technique in patients of this age barring their skeletal age lags their chronologic age. Future studies are indicated on younger patients in whom all-epiphyseal technique would be more likely implemented.

## Conclusions

This study provides an initial examination and reference for utilizing MRI to measure an intact PCL to predict ACL graft size for pediatric ACL reconstruction and also demonstrates statistically significant differences between tunnel lengths based on all epiphyseal drilling method chosen. This is the first study to develop a method for calculating native ACL coronal and sagittal width from an intact PCL in the pediatric population and to propose using this method as a guide for determining ACL graft size.

## Abbreviations

ACL: Anterior cruciate ligament
ACL:PCL ratio: Anterior cruciate ligament: Posterior cruciate ligament ratio
CONSORT: Consolidated Standards of Reporting Trials
EMR: Electronic medical record
IBM: International Business Machines
IRB: Institutional review board
MRI: Magnetic resonance imaging
*p*-value: Probability value
PACS: Picture Archiving and Communication System
PCL: Posterior cruciate ligament
R: Pearson’s correlation
SD: Standard deviation
SPSS: Statistical Package for the Social Sciences.
